# Neurofilament light protein in CSF and blood is associated with neurodegeneration and disease severity in Huntington’s disease R6/2 mice

**DOI:** 10.1038/s41598-017-14179-1

**Published:** 2017-10-26

**Authors:** Rana Soylu-Kucharz, Åsa Sandelius, Marie Sjögren, Kaj Blennow, Edward J. Wild, Henrik Zetterberg, Maria Björkqvist

**Affiliations:** 1 Wallenberg Neuroscience Center, Department of Experimental Medical Sciences, Brain Disease Biomarker Unit, Lund University, Lund, Sweden; 2 Institute of Neuroscience and Physiology, Department of Psychiatry and Neurochemistry, The Sahlgrenska Academy at University of Gothenburg, Mölndal, Sweden; 3 Clinical Neurochemistry Laboratory, Sahlgrenska University Hospital, Mölndal, Sweden; 4 UCL Institute of Neurology, Queen Square, London, UK; 5 UK Dementia Research Institute, London, UK

## Abstract

There is an unmet need to reliably and non-invasively monitor disease progression in preclinical Huntington’s disease (HD) models. As a marker of axonal damage, neurofilament light chain (NfL) has been suggested a marker for neurodegeneration. NfL concentrations in blood and CSF were recently shown to have prognostic value for clinical HD progression and brain atrophy. We therefore hypothesized that CSF and blood NfL concentrations could be useful preclinical HD markers, reflecting underlying pathology. To test our hypothesis we utilized the R6/2 mouse model of HD and measured NfL concentrations in CSF and serum using the ultrasensitive Single molecule array (Simoa) platform. In addition, we assessed HD mouse disease characteristics. We found robust increases of NfL in CSF and serum in R6/2 mice compared to wild-type littermates. CSF and serum concentrations of NfL were significantly correlated, suggesting similar marker potential of serum NfL. CSF and serum concentrations of NfL correlated with disease severity, as assessed by striatal volume and body weight loss. We here provide evidence that CSF and blood NfL concentrations can be used as accessible and reliable pre-clinical HD markers. This will be of potential use for monitoring HD mouse model disease progression and evaluating preclinical disease-modifying treatment response.

## Introduction

Huntington’s disease (HD) is an inherited neurodegenerative disorder caused by a CAG triplet repeat expansion in the gene encoding huntingtin^1^. Clinical HD is characterised by a variable phenotypic expression of motor, cognitive and psychiatric symptoms^2^, middle age onset and slow disease progression^3^.

Mouse models of HD recapitulate many clinical HD features, such as progressive loss of memory and motor dysfunction. In addition, human and mouse HD share similar transcriptional profile changes and neuronal atrophy^4^. The transgenic R6/2 mouse model, first developed in 1996^5^, displays rapid and reproducible progression of HD-like symptoms, such as motor dysfunction, weight loss and striatal volume reduction^5,6^. Therefore, the R6/2 mouse model is widely used for investigating underlying disease pathological processes as well as for assessing therapeutic strategies^7^.

Neurofilaments are neuron-specific protein components of the axonal cytoskeleton^8^. NfL concentration in cerebrospinal fluid (CSF) has been shown to be a marker of axonal injury in several neurological disorders^9–11^. Recent evidence, published by Byrne *et al*., illustrates the biomarker potential of NfL in HD, showing that plasma and CSF levels of NfL are higher in HD subjects prior to disease onset, and that baseline NfL level has independent prognostic power for subsequent disease onset, progression and brain atrophy^12^.

With the future holding promise for disease modifying strategies^13^, there is an unmet need for translational biofluid biomarkers with validity in preclinical models and human patients, to monitor disease progression and treatment response in laboratory conditions. Neurofilament light chain (NfL) concentrations in body fluids have been shown to reflect pathology and symptoms in proteinopathy mouse models of neurodegeneration^14^ but have never been examined in an HD animal model before.

Here, we quantified NfL in CSF and blood in R6/2 mice using the ultra-sensitive immunoassay, Single molecule array (Simoa)-based immunoassay technology^15–17^. We found that NfL was elevated in R6/2 mice in both fluids; its level was significantly associated with measures of clinical and pathological severity; and CSF NfL level increase precedes HD pathology.

## Results

### NfL concentrations are increased in R6/2 mouse serum and CSF

**Figure 1:**
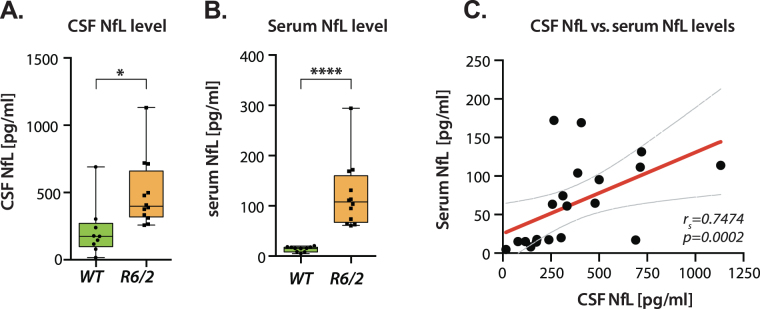
Increased NfL level in CSF and serum in R6/2 HD mice at 18 weeks. (**A**) R6/2 mice exhibited increased neurofilament light chain (NfL) levels in CSF (Student’s t-test, p = 0.0142, n = 9–12/per group) and (**B**) serum (Student’s t-test, p < 0.0001, n = 10–12/per group). (**C**) Significant association between CSF and serum NfL (p = 0.0002). Data in **A,B** are represented as box plots, with boxes representing 25–75 percentile, horizontal lines are median, and whiskers extend to minimum to maximum values. Curved lines represent 95% confidence bands for the linear fit. r_*s*_ = Spearman’s correlation coefficient.

Given that an increased concentration of NfL was detected just before clinical onset in HD patients^12^, we measured CSF and serum levels of NfL in 18 weeks old R6/2 mice and their WT littermates. In both serum and CSF, NfL concentrations were significantly increased in R6/2 mice. Compared to wild-type littermates (Fig. 1A and B), HD mice exhibited ~9–fold increase of NfL in serum (p < 0.0001; 121.3 ± 19.16, n = 12 and 13.79 ± 1.591, n = 10) and ~2.3 –fold increase of NfL in CSF (p = 0.0142; 491.1 ± 73.04, n = 12 and 215.9 ± 65.61, n = 9). Even the lowest serum and CSF NfL concentration in the study (17.04 pg/mL) were still detectable and above the lower limits of detection and quantification of the assay. As with the findings of Byrne *et al*. in humans, we found a significant positive association between matched CSF and serum NfL levels (r_s_ = 0.7474; p = 0.0002) (Fig. 1C). The CSF:serum NfL ratio was approximately 5 in R6/2 and 14 in WT, with significant difference between R6/2 and WT (p = 0.0074), in keeping with CNS origin for the NfL detected in serum.

### Severity of R6/2 phenotype correlates with CSF and serum NfL level increase

**Figure 2:**
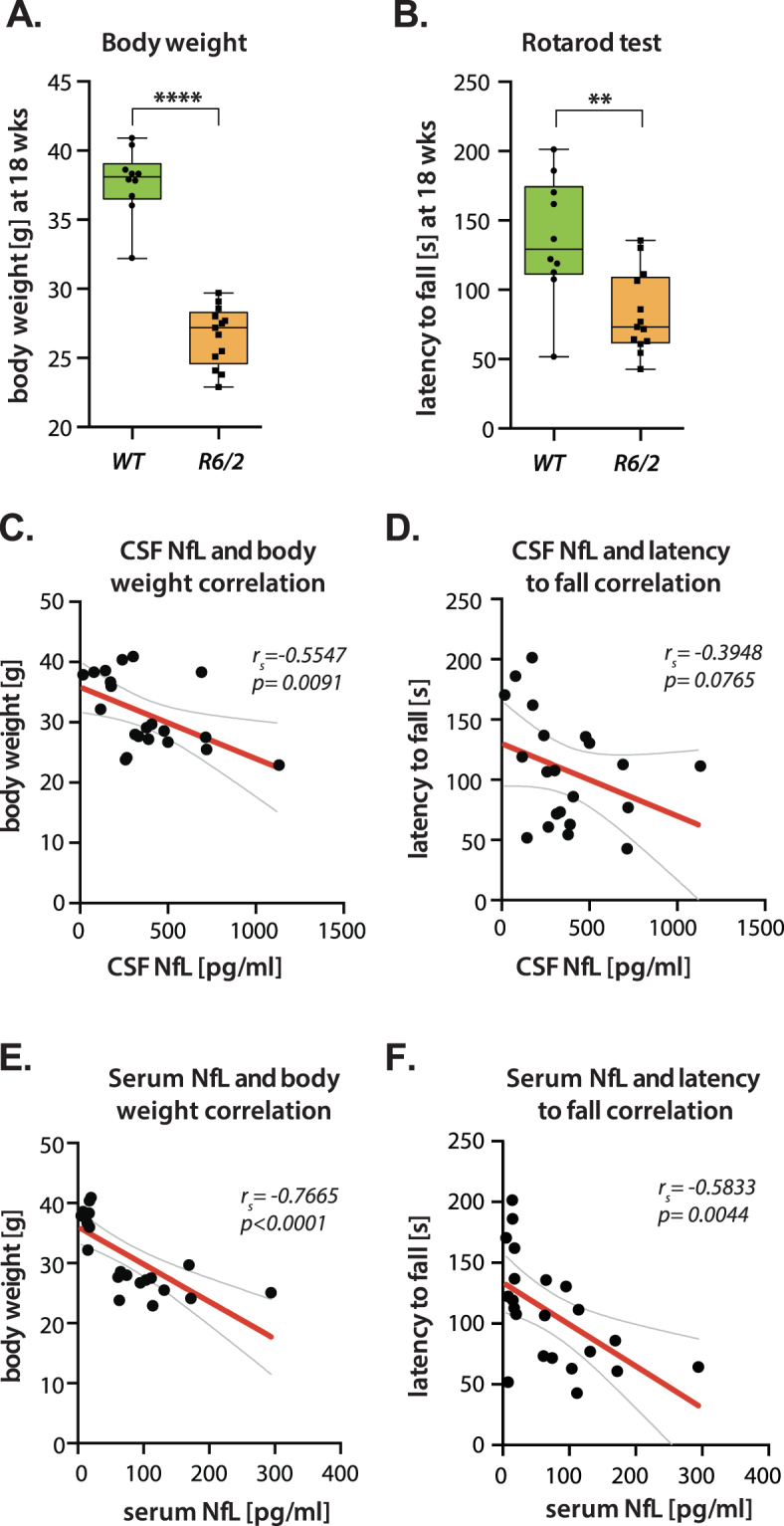
Severity of HD phenotype correlates with CSF NfL level increase. (**A**) HD mice exhibited decreased body weight compared to their WT littermates at 18 weeks (Student’s t-test, p < 0.0001, n = 10–13/per group). (**B**) Behavior rotarod test demonstrating impaired motor function in HD mice. Motor deficit in R6/2 mice is evident by decreased latency to fall from rotarod (Student’s t-test, p = 0.002, n = 10–13/per group) compared to WT littermates. (**C**) CSF NfL was negatively associated with body weight (p = 0.0091), but (**D**) was not significantly associated with motor function (p = 0.0765). Serum NfL level was negatively associated with (**E**) body weight (p < 0.0001) and (**F**) motor function (p = 0.0044). Data in **A**-**B** are represented as box plots, with boxes representing 25–75 percentile, horizontal lines are median, and whiskers extend to minimum to maximum values. Curved lines represent 95% confidence bands for the linear fit. r_*s*_ = Spearman’s correlation coefficient.

As expected, and consistent with the manifestations of the disease in humans, 18 week R6/2 mice (approximately equivalent to the mid-stage of HD) exhibited body weight decrease (p < 0.0001; 26.6 ± 0.6, n = 13 and 37.7 ± 0.8, n = 10) and motor dysfunction assessed using rotarod (p = 0.002; 82.8 ± 8, n = 12 and 137 ± 14, n = 10) compared to their WT littermates (Fig. 2A and B). We found a significant negative association between CSF NfL concentration and body weight, such that higher CSF NfL level was associated with lower body weight (r_s_ = −0.5547, p = 0.0091) (Fig. 2C). No significant association was seen between CSF NfL and motor function deficit on the (r_s_ = −0.3948; p = 0.0765) (Fig. 2D). Strikingly, serum NfL levels were significantly negatively associated with body weight (r_s_ = −0.7665; p < 0.0001) (Fig. 2E) and motor function (r_s_ = −0.5883; p = 0.0044) (Fig. 2F).

### NfL level increase is associated with R6/2 mouse neuropathology

**Figure 3:**
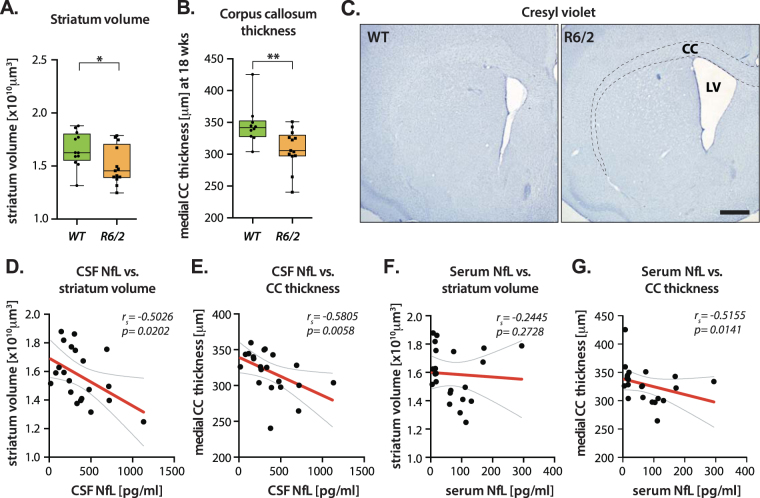
CSF NfL level increase is correlated with histopathological phenotype in HD. (**A**,**B**) Stereological assessments of (**A**) dorsal striatum volume and (**B**) medial corpus callosum thickness showed a pathological reduction in brain matter in R6/2 compared to WT mice. (**C**) Cresyl violet staining of representative brain sections demonstrating reduction in dorsal striatum and medial corpus callosum in R6/2 HD mice. (**D**) CSF NfL level was negatively associated with striatal volume (p = 0.0202) and (**E**) corpus callosum thickness (p = 0.0058). (**F**) There was no significant association between serum NfL and striatal pathology (p = 0.2728). (**G**) Medial corpus callosum thickness was significantly correlated with serum NfL concentrations (p = 0.0141). Data in A-B are represented as box plots, with boxes representing 25–75 percentile, horizontal lines are median, and whiskers extend to minimum to maximum values. Curved lines represent 95% confidence bands for the linear fit. CC: Corpus callosum; r_*s*_ = Spearman’s correlation coefficient. Scale bar is 500 μm.

One of the most early, prominent and progressive changes in HD brain is volumetric loss in the striatum^18–20^. Stereological assessment of cresyl violet stained brain sections showed ~10% reduced dorsal striatal volume (p = 0.0374) and ~11% reduced white matter, assessed as medial corpus callosum thickness (p = 0.0096) in R6/2 mice (Fig. 3A and B). Higher CSF NfL values were associated with greater loss of striatal volume (r_s_ = −0.5026; p = 0.0202) and white matter thickness (r_s_ = −0.5805; p = 0.0058) (Fig. 3D and E). On the other hand, serum NfL concentrations were not correlated with striatal volume loss (r_s_ = −0.2445; p = 0.2728) but significantly correlated with reduction in medial corpus callosum thickness (r_s_ = −0.5155; p = 0.0141) (Fig. 3F and G).

### CSF NfL level increase precedes HD pathology

**Figure 4:**
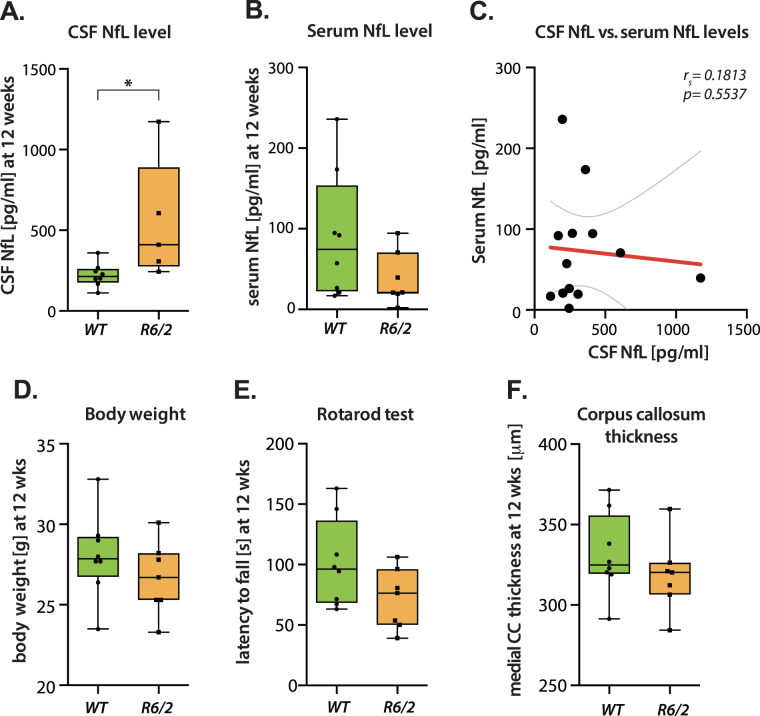
Increased NfL level in CSF in R6/2 HD mice at 12 weeks. (**A**) HD mice exhibited increased neurofilament light chain (NfL) levels in CSF at 12 weeks of age (Student’s t-test, p = 0.0326, n = 5–8/per group). (**B**) Serum NfL concentrations were not different in R6/2 mice compared to WT littermates (Student’s t-test, p = 0.133, n = 7–8/per group). (**C**) There was no association between CSF and serum NfL (p = 0.5537) concentrations. (**D**–**F**) R6/2 mice did not exhibit changes in (**D**) body weight (Student’s t-test, p = 0.3, n = 7–8/per group), (**E**) motor function (Student’s t-test, p = 0.094, n = 7–8/per group) and (**F**) medial corpus callosum thickness (Student’s t-test, p = 0.326, n = 7–8/per group) compared to their WT littermates. Data in **A,B** and **D–E** are represented as box plots, with boxes representing 25–75 percentile, horizontal lines are median, and whiskers extend to minimum to maximum values. Curved lines represent 95% confidence bands for the linear fit. CC: Corpus callosum; r_*s*_ = Spearman’s correlation coefficient.

We assessed NfL levels in 12 week-old R6/2 mice, which in our R6/2 mouse colony corresponds to late premanifest HD. CSF NfL concentrations were significantly increased in R6/2 mice compared to WT littermates with ~2.5–fold increase in R6/2 mice (p = 0.0326; 548.6 ± 168, n = 5 and 222.5 ± 25.96, n = 8), with no significant difference in NfL serum levels (p = 0.1333; 38.37 ± 12.43, n = 7 and 89.79 ± 27.88 n = 8) (Fig. 4A and B). At this timepoint, there was no significant association between CSF NfL and serum NfL levels (p = 0.5537). This CSF increase occurred before manifestation of HD pathology, as there was no significant difference in body weight (p = 0.3; 28.05 ± 0.9325, n = 8 and 26.67 ± 0.8521, n = 7), motor performance (p = 0.0942; 101.5 ± 12.95, n = 8 and 71.76 ± 9.478, n = 7) or corpus callosum thickness (p = 0.326; 331.5 ± 9.007, n = 8 and 318.7 ± 8.616, n = 7) (Fig. 4D–F).

In 4–6 week old mice, which correspond to early premanifest HD, CSF NfL concentrations were similar to WT (p = 0.2319; 189.9, n = 7 and 134.3, n = 8), and there was no significant difference in NfL levels in serum (p = 0.0676; 36.55, n = 10 and 153.4, n = 8) (Supplementary Figure 1A and B). There was no significant correlation between CSF NfL and serum NfL levels (p = 0.3607), and both 4–6 weeks old R6/2 and WT mice had similar body weight (p = 0.1667; 24.1, n = 8 and 22.3, n = 10) (Supplementary Figure 1C and D).

## Discussion

Release of neurofilaments, the major constituents of the neuronal cytoskeleton, has now been shown a feature of many neurological disorders^21,22^. Byrne and co-workers recently presented convincing data on NfL concentrations in CSF and plasma with striking prognostic power for progression in clinical HD^12^. For the first time, we here validate this strategy in an animal model of HD used in drug design strategies, showing that monitoring NfL levels in CSF and plasma may be a marker of relevance in pre-clinical studies.

The R6/2 mouse is a well-characterized HD mouse model mimicking human HD pathology^5^. R6/2 mice are widely used for investigating underlying disease pathological mechanisms and for evaluating disease modification strategies^4^. Here we found that in R6/2 mice, NfL concentrations in CSF and serum are increased in comparison to wild-type mice. We further show that NfL concentrations in serum are correlated with CSF concentrations, in line with human HD findings^12^, with the overall level of NfL 9-fold higher in CSF than in serum, affirming the likely CNS origin of the serum NfL, and suggesting that in mice, serum NfL concentrations could be used to estimate CSF levels. Our findings are in line with recent studies describing the potential of NfL levels as pre-clinical murine biomarkers of neurodegeneration^14,23,24^. Bacioglu and coworkers showed in 2016 that NfL levels in blood and CSF to be increased in mouse models of α-synucleinopathies, tauopathy, and β-amyloidosis^14^. Furthermore, in the present study, serum and CSF NfL concentrations were associated with body weight loss and motoric dysfunction, both robust features that present progressively in R6/2 mice.

Interestingly, the fold NfL difference between R6/2 and WT animals within each fluid was greater in serum than in CSF (9-fold versus 2.3-fold respectively). NfL is a relatively dynamic marker whose current concentration is likely determined by the balance of axonal damage and repair, diffusion into and removal from CSF, and diffusion and removal from blood. The rate at which changes in brain pathology are reflected in the CSF and plasma level remains incompletely understood. Assuming the NfL is originating from the CNS, this finding may indicate that at 18 weeks, as the mice approach end-stage, the release of NfL from CNS neurons is slowing, while an accrued buildup of NfL in serum from earlier in the disease course has yet to be removed from the periphery. This may also explain why CSF but not serum NfL levels were associated with brain volume measurements. This dynamic variation in NfL may also explain our finding that the CSF:serum NfL ratio was here approximately 5 in R6/2 and 14 in WT. Blood-brain barrier variations^25^ and peripheral differences in NfL metabolism may also contribute to these findings.

Neuronal atrophy is widespread in R6/2 mice^5^ and here we showed that concentrations of CSF NfL were correlated with reduced striatal volume and white matter loss, in line with human HD findings^12^. In this study we used histological measures to assess striatal volume and white matter loss. Striatal volume reflects a small proportion of total brain atrophy and it is possible that this results in our finding that only CSF NfL and not serum NfL concentration correlates with striatal atrophy. Further, this is similar to findings presented in Byrne *et al*., where plasma NfL was more closely associated with whole brain than with striatal atrophy.

Notably, CSF NfL concentrations were increased in 12 week old R6/2 mice, before manifestation of typical HD pathology hallmarks, whereas there was no change in serum levels of NfL. These findings are consistent with findings in other neurodegenerative diseases, i.e. α-synucleinopathies and tauopathies, where an increase in CSF NfL concentrations is present before the onset of neurological signs detetected^14^. Given that blood NfL levels are suggested to be derived mainly from CNS^14,26^, we hypothesise that assessment of CSF NfL could be a more reliable biomarker of disease progression, especially at the very early pre-symptomatic stages of HD. Whether this CSF-plasma disconnect in a very rapidly progressing mouse model will be replicated in human HD mutation carriers in very early premanifest HD remains to be seen, but it is certainly of interest in understanding the dynamics of NfL release and informing the design of animal model work.

We conclude that NfL concentrations in serum and CSF are potential preclinical murine markers of HD progression. Our findings form the foundation for establishing the value of NfL as a translational as well as prognostic biomarker for HD. There is a pressing need to characterize CSF and serum NfL across animal models of HD, throughout the disease spectrum, and to investigate whether and how it responds to neuroprotective treatments at different stages including before the onset of symptoms.

## Methods

### Animals

All experimental procedures performed on mice were carried out in accordance with the approved guidelines in the ethical permit approved by The Malmö/Lund Animal Welfare and Ethics Committee (ethical permit number: M135-14). The experiments were carried out on 4–6, 12 and 18 weeks old male R6/2 mice and their WT littermates. The animals were kept at 12 hours night/day cycle with free access to water and normal chow diet. Mice were obtained through crossing heterozygous R6/2 males with WT females. Genotyping was performed as described previously^5^. The R6/2 mice used in this study had a CAG repeat size range between 273–285, resulting in a slower disease progression compared to R6/2 mice with 150 CAGs. 18 week old R6/2 mice correspond to mid stage disease and 12 week old R6/2 mice correspond to late premanifest disease^27^.

### NfL Simoa measurements

Mouse CSF and serum NfL concentration was determined using the in-house Simoa NfL assay which has been described in detail previously^16^. Briefly, paramagnetic carboxylated beads (Quanterix Corp, Boston, MA, USA) was coated with a mouse anti-neurofilament light antibody (UD1, UmanDiagnostics, Umeå, Sweden) and incubated 35 minutes with sample and a biotinylated mouse anti–neurofilament light antibody (UD2, UmanDiagnostics) in a Simoa HD-1 instrument (Quanterix). The bead-conjugated immunocomplex was thoroughly washed before incubation with streptavidin-conjugated β-galactosidase (Quanterix). After additional washes, resorufin β-D-galactopyranoside (Quanterix) was added and the immunocomplex was applied to a multiwell array designed to enable imaging of every single bead. The average number of enzymes per bead (AEB) of samples was interpolated onto the calibrator curve constructed by AEB measurements on bovine NfL (UmanDiagnostics) serially diluted in assay diluent. Samples were analyzed using one batch of reagents and animal treatment information was blinded to the one performing the analysis. The average repeatability of the assay was assessed by measurements of quality control samples and the coefficient of variation was 7.5% for a sample with a mean NfL concentration of 54.2 pg/ml, and 4.5% for a sample with a mean NfL concentration of 5.6 pg/ml. The limit of detection (LOD), determined as the mean blank signal +3 SD for the Simoa NfL assay was 0.3 pg/mL and the lower limit of quantification (LLOQ) determined as the mean blank signal +10 SD was 2.7 pg/mL when compensated for a four-fold sample dilution.

### Rotarod test

Rotarod test was used to monitor motor coordination and balance^28^. Mice were transported in their home cages to the behavioral testing room and were allowed to acclimate to the room for at least one hour prior to testing. Mice were trained on the rotarod (Rotamex 4/8, Rota Rod Columbus Instruments, Ohio, USA) for 5 min in fixed speed (4 rpm), prior to 3 accelerating tests (5 minutes from 4 rpm to 40 rpm), with 15 minutes rest between each test. Mice were allowed to rest one hour between training and first trial session. Latency to fall (s) was calculated as the mean of the 3 tests for each mouse. The rotarod apparatus was cleaned with 30% ethanol between each trial.

### Serological analysis

Cerebrospinal fluid (CSF) samples were collected from 4^th^ ventricles of R6/2 and WT mice under terminal dose of anesthesia. First, heads of the mice were mounted on a stereotaxic frame, and the skin and muscle tissue was dissected to the expose the dura mater above the cisterna magna. CSF samples were collected using glass capillaries (outer diameter = 80 μm) connected to 1 ml syringe through polyethylene tubing.

Blood was collected from the right heart ventricle. To prevent applying large suction force, a wide-bore needle (18 G/40 mm) was used. The blood samples were stored at room temperature for 30 minutes, and then centrifuged at ~2500 × g for 15 minutes. Following the centrifugation supernatant (serum) was collected. CSF and blood serum samples were frozen in dry ice and stored at −80 °C until further use.

### Immunohistochemistry

Mice were perfused transcardially under terminal sodium pentobarbital anesthesia (Apoteksbolaget) with saline and subsequently with ice cold 4% paraformaldehyde (PFA) for 10 min (at the rate of 10 ml/min). Brains were extracted and placed in 4% PFA solution for 24 hours at 4 °C for post-fixation, then transferred to 25% sucrose solution at 4 °C for ~24–36 hours. Brains were sectioned in coronal plane (30 μm sections, six series per animal) on dry ice and the sections were stored at −20 °C in an antifreeze solution (30% glycerol, 30% ethylene glycol in phosphate buffer).

Free-floating brain sections were rinsed 3 × 10 min with 0.05 M tris (hydroxymethyl) aminomethane (Tris) -buffered saline (TBS) and were mounted on gelatine-coated glass slides. The slides were passed through xylen for delipidation and a decreasing series of alcohol (100%, 95%, and 70%, for 1 min each) and then rehydrated in the distilled water bath. Following that, sections were exposed to 0.5% cresyl violet in 10% acetic acid solution for 3 minutes to stain the cell bodies. Stained specimens were then dehydrated with a series of alcohol solutions (70%, 95%, 100%, each for 2 min) and secured with coverslips using Depex (Sigma-Aldrich).

### Stereological analyses

Stereological analyses of striatum volume were carried out with Nikon 80i microscope equipped with 4X objective (NewCast Module in VIS software; Visiopharm A/S, Horsholm); X–Y motorized stage (Märzhauser, Wetzlar) with a high precision linear encoder (Heidenhain, Traunreut). The rostral-caudal boundaries for dorsal striatum volume and corpus callosum thickness measurements were between bregma 1.70 to 0.62 mm for dorsal striatum and bregma 1.10 to −0.10 for corpus callosum thickness. Supplementary Figure 2 shows the boundaries of brain areas used for dorsal striatum and medial corpus callosum thickness measurements.

### Statistical analysis

Statistical analyses were performed using Prism 6.0 (GraphPad). Data were tested first for normal distribution with Kolmogorov-Smirnov test, and two-tailed Student’s t-test or Mann-Whitney test was used, accordingly. The association between parameters was examined using Spearman’s correlation analysis and graphs were plotted with curves indicating 95% confidence interval. For all data, mean was provided with SEM in the results section. The data were shown as box plots with boxes representing 25–75 percentile, whiskers denoting minimum to maximum data range and horizontal line indicating median value. Values for individual mice are plotted on the graphs. In all tests, differences were considered statistically significant for p < 0.05.

## Electronic supplementary material

Supplementary Figure 1 and Figure 2
