# Supplementary material for: Neurofilament light protein in CSF and blood is associated with neurodegeneration and disease severity in Huntington’s disease R6/2 mice
Source: Sci Rep. 2017 Oct 26;7:14114. doi: 10.1038/s41598-017-14179-1 (PMC5658344; doi:10.1038/s41598-017-14179-1)
Supplement: Supplementary file 1 — Supplementary Figure 1 and Figure 2 [file 41598_2017_14179_MOESM1_ESM.pdf]

Neurofilament light protein in CSF and blood is associated with neurodegeneration and disease severity in Huntington's disease R6/2 mice

Rana Soylu-Kucharz[1], Åsa Sandelius[2], Marie Sjögren[1], Kaj Blennow[2,3], Edward Wild[4], Henrik Zetterberg[2,3,4,5], Maria Björkqvist[1]

[1]Wallenberg Neuroscience Center, Department of Experimental Medical Sciences, Brain Disease Biomarker Unit, Lund University, Lund, Sweden

[2]Institute of Neuroscience and Physiology, Department of Psychiatry and Neurochemistry, The Sahlgrenska Academy at University of Gothenburg, Mölndal, Sweden

[3]Clinical Neurochemistry Laboratory, Sahlgrenska University Hospital, Mölndal, Sweden

[4]UCL Institute of Neurology, Queen Square, London, UK

[5]UK Dementia Research Institute, London, UK

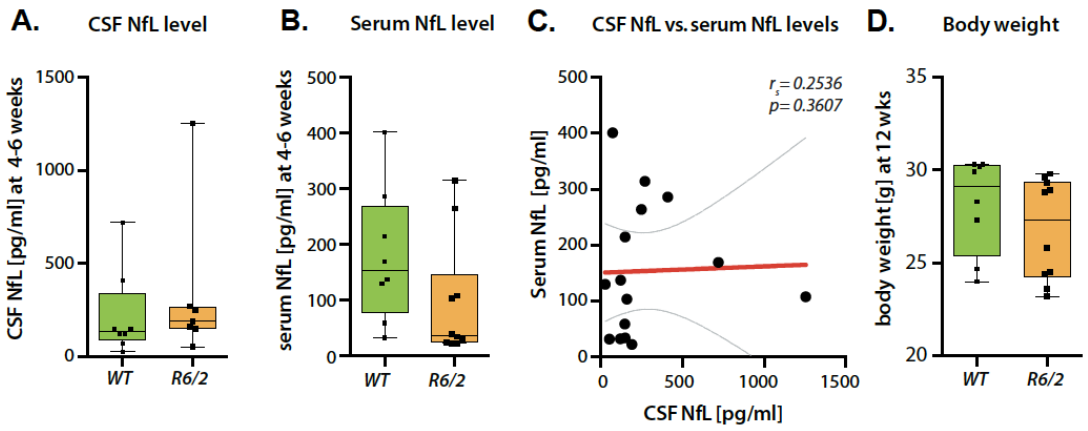

Supplemental Figure 1: No difference in CSF and serum NfL levels between R6/2 and WT mice at 6 weeks. (A) CSF (Mann-Whitney test, p=0.212, n=7-8/per group) and **(B)** serum NfL concentrations (Mann-Whitney test, p=0.068, n=8-10/per group) in R6/2 and WT littermates. **(C)** There was no correlation between CSF and serum NfL levels (p=0.3607). **(D)** R6/2 mice exhibit similar body weight compared to WT mice (Mann-Whitney test, p=0.167, n=7-8/per group). r_s=Spearman's correlation coefficient. Data in A-B and D-E are represented as box plots, with boxes representing 25–75 percentile, horizontal lines are median, and whiskers extend to minimum to maximum values.

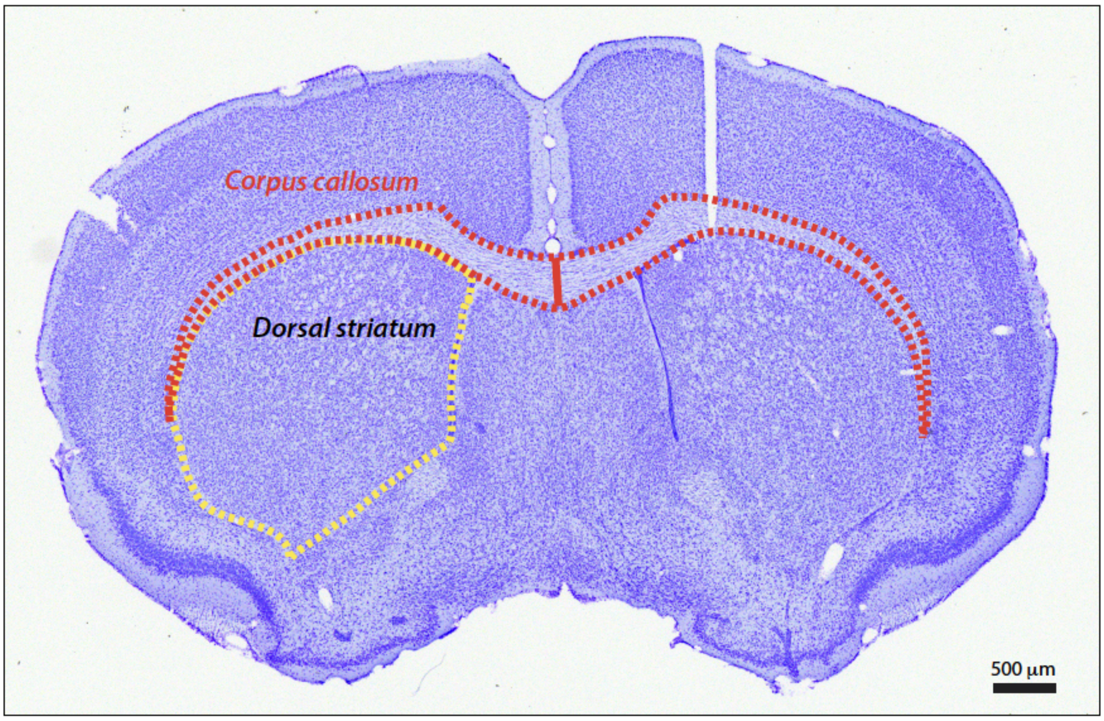

Supplemental Figure 2: A coronal section of cresyl violet-stained mouse brain with yellow and red trace denoting the boundaries of brain areas used for dorsal striatum and corpus callosum thickness measurements, respectively.
